# Heavy metals and hydrocarbon concentrations in water, sediments and tissue of *Cyclope neritea* from two sites in Suez Canal, Egypt and histopathological effects

**DOI:** 10.1186/s40201-015-0171-5

**Published:** 2015-03-10

**Authors:** Hesham M Sharaf, Abdalla M Shehata

**Affiliations:** Department of Zoology, Faculty of Science, Zagazig University, Zagazig, Egypt; Chemistry Department, Faculty of Science, Suez Canal University, Al-arish, Egypt

**Keywords:** Heavy metals, Hydrocarbons, Timsah Lake, Suez canal, *Cyclope neritea*

## Abstract

Heavy metals and hydrocarbons are of the most common marine pollutants around the world. The present study aimed to assess the concentration of petroleum hydrocarbons and heavy metals in tissues of the snail *cyclope neritea,* water and sediments from two sites of the study area (Temsah lake and Suez canal) represent polluted and unpolluted sites respectively. The results showed that, the levels of the heavy metals (Pb, Cd, Co, Mg and Zn) in the polluted area have reached harmful limits recorded globally. Lead in water, sediment and tissue of the snail reached to 0.95 ppm, 4.54 ppm and 7.93 ppm respectively. Cadmium reached 0.31 ppm, 1.15 ppm and 3.08 ppm in the corresponding samples. Cobalt was not detected in water, but it reached 1.42 ppm and 10.36 ppm in the sediment and snails tissue respectively. Magnesium in water, sediment and tissue of the snail reached 3.73 ppm, 9.44 ppm and12.6 ppm respectively. Zinc reached 0.11 ppm, 3.89 ppm and 12.60ppm in the corresponding samples. Meanwhile, hydrocarbons in the polluted area (site1) reached 110.10 μg/L, 980.15 μg/g and 228.00 μg/g in water sediment and digestive gland tissues of the snails respectively. Whereas, hydrocarbons in the unpolluted area (site2) were estimated as 14.20 μg/L, 55.60 μg/g and 22.66 μg/g in water, sediment and tissue of the snails respectively.

The combination of histopathological image with monitoring of the metal level in the digestive gland of the present snail provides an important tool for early detection of impending environmental problems and potential public health issues. Petroleum hydrocarbons are toxic to the marine fauna when present above certain limit in the marine water. The major detoxification organ in molluscs is the digestive gland, which has been used as a bioindicator organ for toxicity assessment. The effect of high crude oil on the digestive gland tubules of exposed snails when examined microscopically reveals a series of histological changes which indicates that the cellular compensatory mechanism is activated by hydrocarbons. These changes include vacuolation and presence of pyknotic nuclei.

## Introduction

Adverse anthropogenic effects on the coastal environment include eutrophication, heavy metals, organic, oil spills and microbial pollution. Consequently, levels of contaminants in the marine environment are increasing continuously. In order to establish adequate coastal management programs, it is important to characterize the environment of concern chemically. The extent of contamination can be assessed by measuring pollutant concentration in water, sediments and exposed animal tissues samples. Trace metals can be divided to essential and non essential elements. Essential elements occur naturally in all organisms, essential elements in high doses can be poisonous causing hazardous effects on organisms. Non essential elements do not have any positive effects on organisms and they are harmful already in low doses. They can inhibit an essential element to bind to enzyme and disturb the normal enzymatic function in the body [1-9].

As compared with the open sea, lagoons are more subject to pollution, particularly by heavy metals from industrial, agricultural and urban origin. Near shore sediments are found in a wide variety of environments (bays, lagoons). The water sediments interface is more important to biological fauna as compared with surface sediments, since meiofauna live about the reduced zone in sediment. Therefore, the composition of sediment has a significant influence on the living conditions of marine organisms. The trace metal results were obtained by sediment analysis, unlike sea water analysis, where the detection limit and contamination risks are significantly reduced [9].

It is well known that molluscs accumulate organic and metal pollutants at concentrations several order of magnitude above those observed in the field environment. Fewer studies have been done on gastropod molluscs, some of which are considered as useful biomonitors of certain metals [10]. Most metals are generally concentrated many times within an organism’s soft tissue.

Advancement in technology as well as increase in population have led to environmental concerns relating from indiscriminate dumping of refuse and discharge of industrial effluents, petroleum waste water and crude oil spills replete with most common heavy metals in our environment [7,11].

Histopathological patterns represent a rapid, sensitive, reliable and comparatively inexpensive tool for assessment of stress response to xenobiotics [12-14]. These cascades of stress related responses including histopathology are now increasingly being used as biomarkers of environmental stress since they provide a definite biological endpoint of historical exposure [15-17].

Heavy metal pollution of terrestrial and aquatic ecosystems has long been recognized as a serious environmental concern. This is largely due to their non biodegradability and tendency to accumulate in plants and animals tissues. As a result, metal bioaccumulation is a major route through which increased levels of the pollutants are transferred across food chains, creating public health problems [18-20]. Therefore, it is important to determine the bioaccumulation capacity for heavy metals by certain organisms in order to assess potential risk to human health.

Several authors [9,13,21] have reported the importance of molluscs as good indicators for monitoring heavy metal pollution even through abnormally high environmental concentrations, since heavy metals affect numerous biological processes involved in the development and maintenance of molluscan populations such as feeding, growth, reproduction and general physiological activities [22].

Contamination of the sea with petroleum hydrocarbons, especially in shipping channels and ports, where crude and refined petroleum products are transported in significant quantities of oil entering the water column and sediment. The hepatopancreas of molluscs is the major site of petroleum hydrocarbons (PHC) detoxification. The main route for elimination of hydrocarbon metabolism is through faecal matter [23].

This study aimed to investigate the concentration of Pb, Cd, Co, Mg, Zn and hydrocarbons in water, sediment and soft tissues of *Cyclope neritea* snail from two different sites at Suez Canal, Egypt, map(1). The first site is located in Timsah Lake which receives effluents from many industrial sources site (1) where as the second site is Suez Canal where there is limited industrial or anthropogenic activity site (2).

Moreover, an attempt has been made to elucidate the response of the digestive gland of *Cyclope neritea snails* to heavy metals and PHCs and to identify histopathological biomarkers.

## Materials and methods

**Figure 1:**
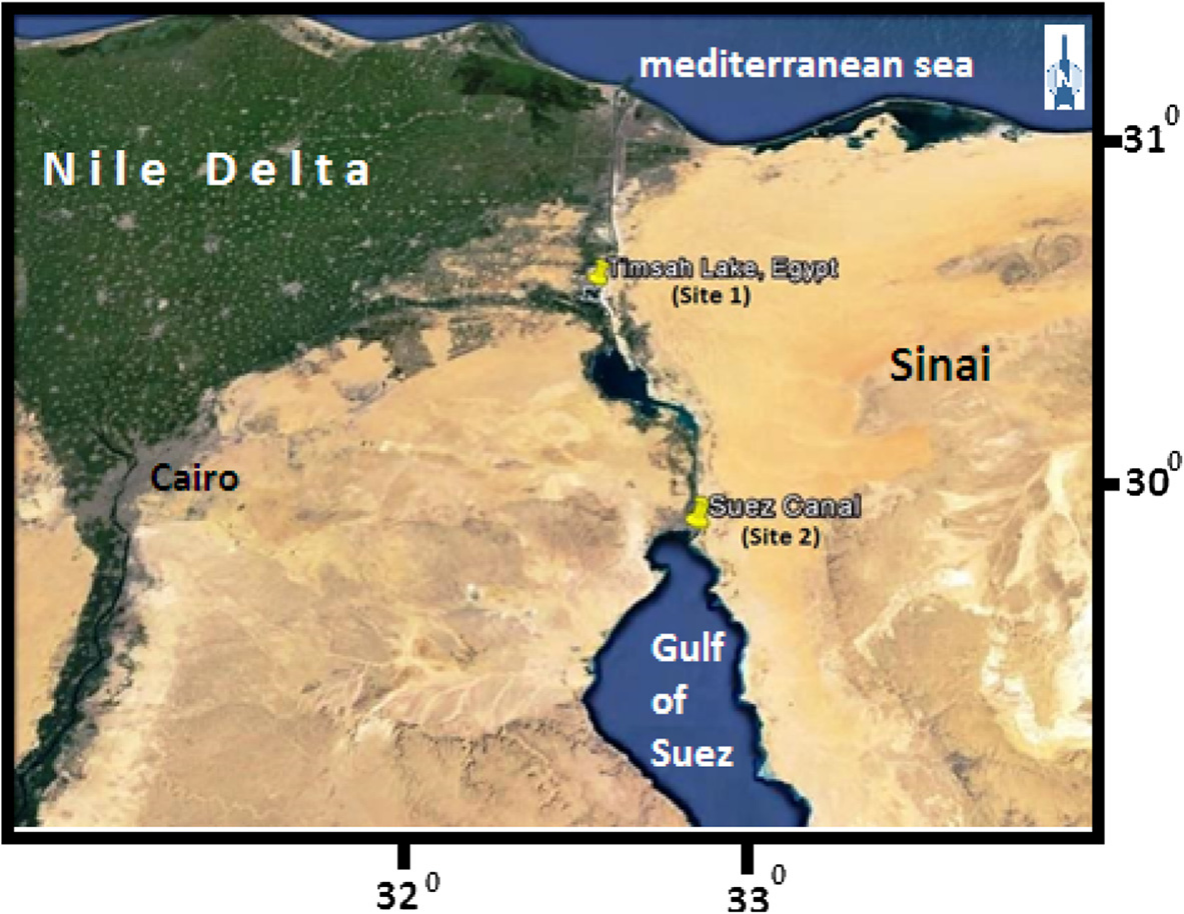
**A map of Suez Canal showing the two investigated sites.** The first site; Timsah Lack and the second site; Suez Canal.

Heavy metal content (Pb, Cd, Co, Mg and Zn) and hydrocarbons were analyzed in the water, sediment and soft tissues of *Cyclope neritea* snails from two different sites at Suez canal, the first was polluted, site1 (Timsah lake) while the second unpolluted site2 (Suez Canal) from the main canal (Figure 1).

### Water and sediment

From both sites water samples were collected directly in a precleaned polyethylene bottles then sealed until analysis, sediment samples were collected using a precleaned PVC corer and immediately kept in polyethylene bags which were sealed and kept in an ice box for further analysis in the laboratory. Sediment samples were washed with bidistilled water and dried at 60°C then ground in a glass mortar and reduced into fine particles.

### Samples collections

Snails were collected from both sites by hand picking. The soft tissues were removed from snail’s shells with a sharp knife and dried at 60°C. The dried tissue was ground into fine powder and stored in a desiccator for further analysis.

### Chemicals and method of analysis

Estimation of trace metals in water and sediment was carried out by suitable volume of water or digestion of one gram of sediment with conc. Nitric acid (HNO3) and conc. Perchloric acid (HClO4) (4:l) and analyzed in Atomic absorption specterophotometers perkin-Elmer, Analyzed TM 300 (USA) at suitable current and wave length for all studied heavy metals [24,25]. The values were expressed in ppm and the standard deviation was calculated. The hydrocarbons in all samples were extracted by Soxhlet instrument and measured by a Spectrofluorophotometer.

### Statistical analysis

SPSS13 for Windows software was used for the statistical analysis of the heavy metal contents in water, sediment and digestive gland tissue of snails at the two investigated sites by using Mann–Whitney test.

For histolopathological examination the digestive gland of *Cyclope neritea* was taken out of their shells and dropped immediately into Alcoholic Bowin’s, fixative then processed in the usual manner for histological investigation and stained with Haematoxylin and Eosin.

## Results

Mean ± SD of the heavy metals at the two investigated sites were tabulated in Table 1. Statistical Mann–Whitney test was applied for all parameters at the two sites.

**Table 1:** **Concentration of heavy metals (ppm) in water, sediment and digestive gland tissue of** ***Cyclope neritea*** **snails (Mean** ± **SD) at the two investigated sites**

**Metal**	**Timsah Lake (site1)**			**Suez Canal (site2)**		
	**Mean** **±** **SD**			**Mean** **±** **SD**		
	**Water**	**Sediment**	**Digestive gland tissue**	**Water**	**Sediment**	**Digestive gland tissue**
**Pb**	0.95 ± 0.04	4.54 ± 0.05	7.93 ± 0.03	0.92 ± 1.04	2.11 ± 0.82	3.93 ± 0.50
**Cd**	0.31 ± 0.04	1.15 ± 0.17	3.08 ± 0.20	0.50 ± 0.02	1.00 ± 0.13	1.72 ± 0.07
**Co**	0.00 ± 0.00	1.42 ± 0.17	10.63 ± 0.21	0.00 ± 0.00	0.67 ± 0.96	2.66 ± 0.25
**Mg**	3.73 ± 0.20	9.44 ± 1.18	12.6 ± 0.96	2.63 ± 0.01	3.92 ± 0.37	5.86 ± 0.15
**Zn**	0.11 ± 0.04	3.89 ± 0.20	12.6 ± 0.96	0.13 ± 0.01	1.33 ± 1.00	4.44 ± 0.80

**Figure 2:**
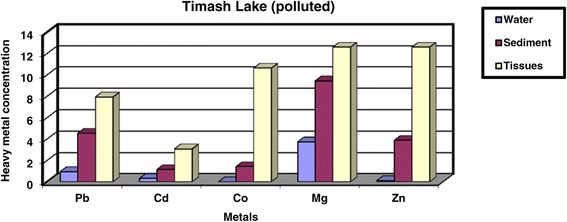
**Concentration of heavy metals (ppm) in water, sediment and digestive gland tissue of** ***Cyclope neritea*** **snails in Timsah Lake (polluted area).**

Heavy metals in Site 1 (Timsah Lake, polluted area) are shown in Table 1, Figure 2. The results revealed that the values of heavy metals in water are 0.95 ± 0.04, 0.31 ± 0.04, 0.00 ± 0.00, 3.73 ± 0.20 and 0.11 ± 0.04 ppm for Pb, Cd, Co, Mg and Zn respectively. Meanwhile, heavy metals values in sediment are 4.54 ± 0.05, 1.15 ± 0.17, 1.42 ± 0.17, 9.44 ± 1.18 and 3.89 ± 0.20 ppm for Pb, Cd, Co, Mg and Zn respectively. Besides, the values of heavy metals in digestive gland tissues are 7.93 ± 0.03, 3.08 ± 0.20, 10.63 ± 0.21, 12.6 ± 0.96 and 12.6 ± 0.96 ppm for Pb, Cd, Co, Mg and Zn respectively.

**Figure 3:**
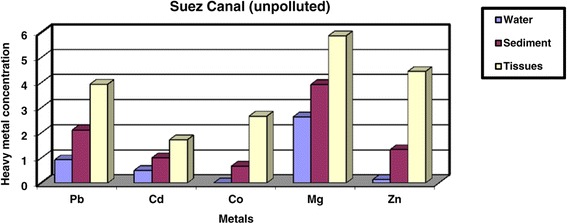
**Concentration of heavy metals (ppm) in water, sediment and digestive gland tissue of** ***Cyclope neritea*** **snails in Suez Canal (unpolluted area).**

On other hand, heavy metals in Site2 (Suez Canal, unpolluted area) are shown in Table 1, Figure 3. The results revealed that, the values of heavy metals in water are 0.92 ± 1.04, 0.50 ± 0.02, 0.00 ± 0.00, 2.63 ± 0.01 and 0.13 ± 0.01 ppm for Pb, Cd, Co, Mg and Zn respectively. Meanwhile, heavy metals values in sediment are 2.11 ± 0.82, 1.00 ± 0.13, 0.67 ± 0.96, 3.92 ± 0.37 and 1.33 ± 1.00 ppm for Pb, Cd, Co, Mg and Zn respectively. And the values of heavy metals in digestive gland tissues are 3.93 ± 0.50, 1.72 ± 0.07, 2.66 ± 0.25, 5.86 ± 0.15 and 4.44 ± 0.80 ppm for Pb, Cd, Co, Mg and Zn respectively.

The Mann–Whitney test was made between the heavy metals concentrations of the digestive gland tissue of the snails and both water and sediment in the two sites, asymptotic significance (2-tailed) = 1.00 and 0.18 respectively was not significant at P < 0.05 level. Also, the Mann–Whitney test was made between the heavy metals concentrations of the digestive gland tissue of the snails in the two sites, asymptotic significance (2-tailed) = 0.047 was significant at P < 0.05 level.

**Figure 4:**
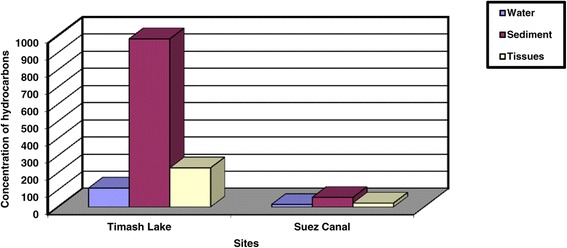
**Concentration of hydrocarbons in water, sediment and digestive gland tissue of** ***Cyclope neritea*** **snails in the study area.**

On the other hand, the concentrations of petroleum hydrocarbons were tabulated in Table 2, Figure 4. The results revealed that concentration of petroleum hydrocarbons are 110.1, 980.15 and 228.00 ug/g for water, sediment and digestive gland tissues respectively in site (1). Meanwhile, they are 14.2, 55.6 and 22.66 ug/g for water, sediment and digestive gland tissues respectively in site (2). The Mann–Whitney test was made between the petroleum hydrocarbons concentrations of the digestive gland tissues and both of water and sediment from the two sits, asymptotic significance (2-tailed) = 0.0499 was significant at P < 0.05 level.

**Table 2:** **Concentration of hydrocarbons in water, sediment and digestive gland tissue of** ***Cyclope neritea*** **snails in the study area**

**Site**	**Water (μg/L)**	**Sediment (μg/g)**	**Digestive gland tissue (μg/g)**
**Timsah Lake**	110.10	980.15	228.00
**Suez Canal**	14.20	55.60	22.66

### Histpathological changes

**Figure 5:**
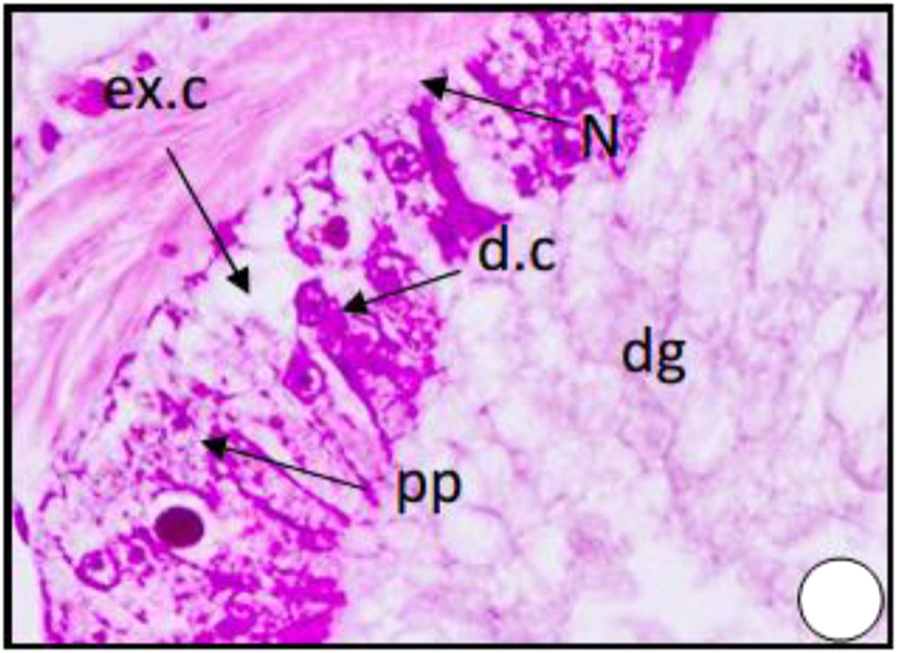
**A photomicrograph of T.S. of digestive gland of** ***Cyclope neritea*** **from the unpolluted site (Suez Canal) showing digestive tubules without hydrocarbon precipitations in the digestive gland cells.** N (Nucleus); dg (digestive gland); d.c (digestive cell); ex.c (excretory cell); PP (hydrocarbon precipitations). X400.

The digestive gland of normal *C. neritea* is a large, tubulo-acinar gland which occupies the greater part of the cavity of the shell spire. The gland is covered by squamous epithelium resting on a thin layer of fibrous connective tissue Figure 5. The digestive gland tubules are lined with simple epithelium. This epithelium consists of two main cell types, digestive cells and secretory cells.

The digestive cells Figure 5 are by far the most numerous elements in the wall of the digestive gland tubules. They are long columnar or cube shaped with domed distal apices and flat bases by which they rest on a very thin basement membrane. They vary greatly in length within the same tubule. The nucleus is basal, usually oval but may be spheroidal or even irregular. Inside the major part of the cell body the cytoplasm is lightly stained and shows various degrees of vaculation and different contents.

The secretory cells Figure 5 are present in much smaller number than the digestive cells. They are shorter pyramidal or cone-shaped, but may sometimes be columnar. They are markedly shorter than the digestive cells and therefore appear wedged in between groups of the digestive cells. Their cytoplasm is basophilic and the body of the excretory cells is usually crowded with numerous spherules (ex.s) of regular form but different sizes. Each excretory cell contains oval or spherical excretory bodies (dark granules) which are usually chromophobic, usually retain their natural colour and appear located inside colourless vacuoles.

**Figure 6:**
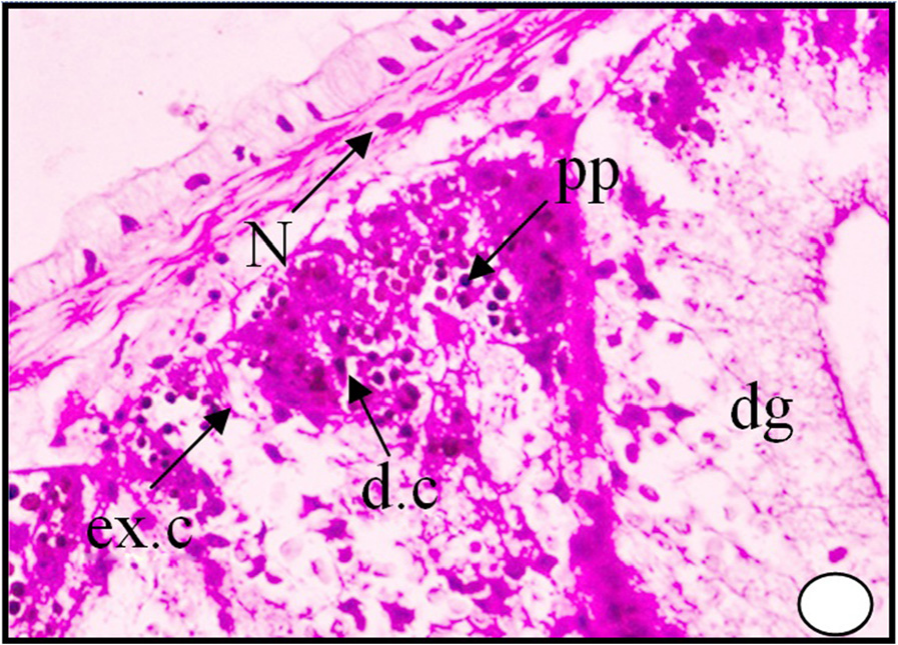
**Digestive tubules T.S. of digestive gland in snail** ***Cyclope neritea*** **from polluted area (Timsah Lake) showing hydrocarbon precipitations in the digestive gland cells.** X200.

**Figure 7:**
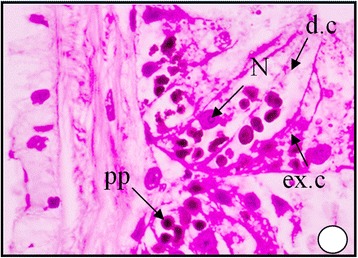
**Higher magnification of Digestive tubules in T.S. of digestive gland of the snail** ***Cyclope neritea*** **from polluted area (Timsah Lake) showing hydrocarbon precipitations in the digestive gland cells.** N (Nucleus); dg (digestive gland); d.c (digestive cell); ex.c (excretory cell); PP (hydrocarbon precipitations); V (vacuolation). X400.

**Figure 8:**
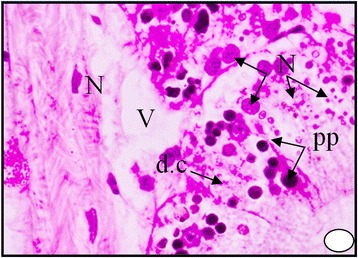
**A photomicrograph of digestive gland of the snail** ***Cyclope neritea*** **from polluted area (Timsah Lake) showing the accumulations of hydrocarbons particles which lead to damaging and vacuolation of most digestive cells of the digestive gland cells.** N (Nucleus); d.c (digestive cell); PP (hydrocarbon precipitations); V (vacuolation). X400.

**Figure 9:**
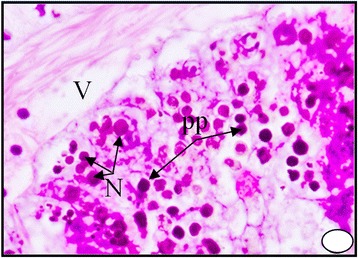
**A photomicrograph of digestive gland of the snail** ***Cyclope neritea*** **from polluted area (Timsah Lake) showing that most of the digestive cells became polygonal and bi and multinucleated and finally degenerated and lysed due to hydrocarbon precipitations.** N (Nucleus); PP (hydrocarbon precipitations); V (vacuolation). X400.

These bodies may represent the final stage in elaboration of the excretory material within the excretory cells. The digestive gland obtained from the polluted area shows an increase in the secretion of the digestive cells, where the cytoplasm becomes more acidophilic and their nuclei start unusual division Figure 6. It can be seen that the dark granules increased greatly in number and most of the digestive cells become completely degenerated and lysing while most of the tubules are damaged Figures 7, 8 and 9. At much higher concentrations of pollutants the digestive gland tubules become mostly damaged, necrotic and the cells take cluster shape and become bi and multinucleated and finally lysed Figures 8 and 9.

## Discussion

This study shed light on some of the environmental factors that might have an impact directly or indirectly on the concentration ability of heavy metals into the bodies of the investigated host. The concentration of water with a wide range of heavy metals has become a matter of great concern over the last few decades [26] and a lot of studies have been published on the heavy metals at all levels of aquatic ecosystem [27-30]. Many authors associated the heavy pollution in water with industrial and municipal discharges. These heavy metals may be taken up by living organisms, deposited in the sediments or remain for some period in the water itself [31,32].

In the present study concentrations of heavy metals in water, sediment and tissues of molluscs from two sites of Suez Canal were determined. The highest concentrations for all studied heavy metals were recorded at site (1) when compared with site (2). The differences in heavy metals concentration between site 1 and site 2 might be attributed to the highly discharge of mixture of industrial, municipal and agricultural drains into site (1), this opinion agrees with [33,34]. Several authors reported that the variation in heavy metals concentration in water might be attributed to the contaminated sediment; these sediments reflect the quality of water current and form the major repository of heavy metals in aquatic system. They added that the rate of accumulation depends mainly on the environmental parameters. Therefore, sediments can be used to detect the presence of contamination that does not remain soluble after the discharge into water [35,36].

The data of the heavy metals in the present work revealed that there is a highly significant (P < 0.01) difference between the two sites, the increase in values of site 1 may be attributed to a number of factors such as industrial effluents, agricultural drainage and waste municipal [31]. Water of the two sites showed higher concentrations of Lead (0.95, 0.92 ppm); Cadmium (0.31, 0.50 ppm), Cobalt (0.00, 0.00 ppm); Manganese (3.73, 2.63 ppm); and Zinc (0.11, 0.13 ppm). Meanwhile, sediment of the two sites showed higher concentrations of Lead (4.54, 2.11 ppm); Cadmium (1.15, 1.00 ppm), Cobalt (1.42, 0.67 ppm); Manganese (9.44, 3.92 ppm); and Zinc (3.89, 1.33 ppm), Also, heavy metals concentration in the digestive gland tissues of *C. neritea* snail of the two sites showed higher concentrations of Lead (7.93, 3.93 ppm); Cadmium (3.08, 1.72 ppm), Cobalt (10.63, 2.66 ppm); Manganese (12.6, 5.86 ppm); and Zinc (12.6, 4.44 ppm), these finding are in agreement with [37] where they determined the levels of heavy metals Lead (Pb), Cobalt (Co), Manganese (Mg), Zinc (Zn) and Cadmium (Cd) in coastal water, sediment and soft tissues of the gastropod limpet *Patella caerulea* and the bivalvae *Barbatus barbatus* from seven different stations in the western coast of the Gulf of Suez. The highest accumulated metals were Fe, Zn and Mn in both *Patella caerulea* than *Barbatus barbatus*. In the present study, metals such as Pb, Mg and Zn, exhibited modreate concentrations. Cadmium and Cobalt metals were below the detectable limit at both sites.

In concordance with the present study, several relevant studies have been made earlier. The heavy metal accumulation in the gastropod *Cerithium scabridium* from Kuwait coast has been analyzed [38] where the concentration of the cadmium in the gills ranged between 7.06 ppm and 0.09 ppm, which is comparatively lower than that in the present study. The concentrations of the analyzed heavy metals exhibited variations in water, sediments and tissues of the studied animal from both sites.

The assessment and description of toxicants on organisms is attracting the interest of the researchers working with marine organisms [2,9,39]. Accumulated hydrocarbons were found to interact with cellular hydrocarbons leading to different histopathological lesions with the highest necrosis index of cells [18]. Cellular and subcellular histopathological changes have proved to be reliable biomarkers in toxicological assays [20]. In the present study, the accumulations of hydrocarbons lead to damaging and vacuolation of most digestive cells, where they became polygonal and bi and multinucleated and finally degenerated and lysed. These results agree with those of [2,17,40-42].

Further studies are still needed to investigate the effect of heavy metal contamination on the biochemical parameters and fine structure to evaluate their potentiality as sentinel organisms to heavy metal contamination in aquatic habitats.
